# Supporting Healthy Aging in the Era of AI: Practical Insights for Aging and Technology Research

**DOI:** 10.1093/geroni/igaf122.1381

**Published:** 2025-12-31

**Authors:** Jane Chung

**Affiliations:** Emory University, Atlanta, Georgia, United States

## Abstract

As artificial intelligence (AI) and digital technologies rapidly evolve, there is a growing opportunity to leverage these innovations to support healthy aging in older adults. However, translating technological advancements into real-world solutions for aging populations requires careful consideration of accessibility, usability, digital literacy, and ethical implications. This panel presentation will share insights from the research on AI-driven and digital technology-based interventions designed to enhance functional assessment, social connectedness, and self-management. Also, the presentation will focus on developing equitable technology solutions and implementing them for sustained use to promote healthy, independent living among community-dwelling older adults including those experiencing health disparities. Drawing on extensive experience in engaging older adults in technology co-design, navigating interdisciplinary collaborations, and securing research funding, this presentation will provide practical guidance for early-career scholars and professionals interested in applying technology to community-based aging research. Key topics will include strategies for designing inclusive and effective digital interventions, overcoming barriers to technology adoption and use among older populations, and fostering meaningful engagement with communities of interest in AI technology development and implementation. By centering the conversation on both innovation and inclusivity, this discussion will equip early-career scholars with actionable strategies to leverage AI for aging research while addressing persistent health disparities.

